# Intersectoral Partnerships Between Local Governments and Health Organisations in High-Income Contexts: A Scoping Review

**DOI:** 10.34172/ijhpm.2024.7841

**Published:** 2024-02-18

**Authors:** Aryati Yashadhana, Karla Jaques, Aulina Chaudhuri, Jennie Pry, Patrick Harris

**Affiliations:** ^1^Centre for Primary Health Care and Equity, University of New South Wales, Sydney, NSW, Australia.; ^2^School of Population Health, University of New South Wales, Sydney, NSW, Australia.; ^3^School of Social Sciences, University of New South Wales, Sydney, NSW, Australia.; ^4^Ingham Institute, Liverpool, NSW, Australia.; ^5^Centre for Health Equity Training, Research and Evaluation, University of New South Wales, Sydney, NSW, Australia.; ^6^South Western Sydney Local Health District, Ingham Institute, Liverpool, NSW, Australia.; ^7^Liverpool Hospital, Liverpool, NSW, Australia; ^8^Healthy Places, Population Health, South Western Sydney Local Health District, Liverpool, NSW, Australia.

**Keywords:** Social Determinants of Health, Local Government, Health Policy, Intersectoral Collaboration, Collaboration

## Abstract

**Background:** Local governments are the closest level of government to the communities they serve. Traditionally providing roads, rates and garbage services, they are also responsible for policy and regulation, particularly land use planning and community facilities and services that have direct and indirect impacts on (equitable) health and well-being. Partnerships between health agencies and local government are therefore an attractive proposition to progress actions that positively impact community health and well-being. Yet, the factors underpinning these partnerships across different contexts are underdeveloped, as mechanisms to improve population health and well-being.

**Methods:** A scoping review was conducted to gain insight into the concepts, theories, sources, and knowledge gaps that shape partnerships between health and local governments. The search strategy followed the Preferred Reporting Items for Systematic Reviews and Meta-Analyses extension for Scoping Reviews (PRISMA-ScR) guidelines and was informed by a critical realist approach that identifies necessary, contingent and contextual factors in the literature. MEDLINE, Scopus, Web of Science, and ProQuest Central databases were searched for studies published between January 2005 and July 2021.

**Results:** The search yielded 3472 studies, after deleting duplicates and initial title and abstract screening, 188 papers underwent full text review. Twenty-nine papers were included in the review. Key themes shaping partnerships included funding and resources; partnership qualities; governance and policy; and evaluation and measures of success. The functional, organisational and individual aspects of these themes are explored and presented in a framework.

**Conclusion:** Given that local government are the closest level of government to community, this paper provides a sophisticated roadmap that can underpin partnerships between local government and health agencies aiming to influence population health outcomes. By identifying key themes across contexts, we provide a framework that may assist in designing and evaluating evidence-informed health and local government partnerships.

## Background

 Local governments are conventionally known to provide their local communities with roads, rates, and garbage services however, they are also responsible for local policy and regulation, particularly through land use planning and community social facilities and services, which impact the health and well-being of their local populations.^1,2^ As a result, partnerships between health agencies and local government are an attractive proposition to progress actions that positively impact community health and well-being. Yet, the way in which partnerships between health agencies and local governments operate in different contexts is not well understood, including if and how they seek to improve population health and well-being.

 By and large, the urban governance focussed literature^3-6^ suggests that the progress and successful implementation of initiatives with local government is subject to two factors: objectives that align with and progress the core business of local government departments (including councils), and the various governance formations of stakeholders that are then created to progress those objectives. Local government has long been identified as a space for health focussed policy collaboration.^7^ Recent interest in health in all policies has focussed attention on local level partnerships between government sectors including but not limited to local government.^8,9^ Recent research has focussed on the consideration of [health] equity in local government policies and programs.^10^ There is some literature^1,11,12^ on the understanding and adoption of health as a concept in local government.^10^ In some jurisdictions local government (municipalities) have become responsible by law for health promotion and prevention. Those municipalities have been investigated for their capability to implement activities to improve health and health equity, including establishing intersectoral collaboration.^13-15^ However, there is limited knowledge specifically identifying factors from interventions to inform theories of change underpinning intersectoral partnerships as the principal mechanism for action.^16^

 This scoping review was undertaken to inform a broader research project, developing a theory of change to underpin an evaluation of partnerships (case studies) between health and local government in Sydney, Australia. Our review aimed to understand what inhibits or enables successful partnerships between local government and health sectors (eg, intersectoral partnerships). Drawing on critical realist approaches,^17^ we aimed to identify factors that shape partnership activities and outcomes across high-income contexts, and collectively map them to identify the underlying mechanisms and wider conditions that drive their success. In doing so we present an evidence-informed conceptual framework that may be of use to partnership planners, facilitators, or evaluators working in health or local government.

## Materials and Methods

 A scoping review approach was taken to gain insight into the main concepts, theories, sources, and knowledge gaps around partnerships between health and local governments.^18^ The search strategy followed the Preferred Reporting Items for Systematic Reviews and Meta-Analyses extension for Scoping Reviews (PRISMA-ScR) guidelines and was developed in consultation with a research librarian.^18^ The following databases were searched for studies published between January 2005 and July 2021: MEDLINE, Scopus, Web of Science, and ProQuest Central. A set of search terms (Box 1) used for each area of interest were compiled. The database search results were imported into a single library in EndNote (Clarivate Analytics, USA) where duplicates were removed. The combined library was imported into Covidence systematic review software (Veritas Health Information, Australia) for title/abstract and full text screening.

**Box 1. Combining Search Term Groups with the Boolean Operator ‘AND’**

**Search #1**

“local government*” OR “provincial government” or “city government*” or “local authority” or “local council*” or “city council*” OR “shire council*” OR municipal* OR “local partnership*”

**Search #2**

policymaker OR policymakers OR initiative* OR “logic model” OR collaboration OR “memoranda of understanding” OR “memorandum of understanding” OR partnership* OR co-production OR co-design OR “capacity building” OR “theory of change” OR intersectoral OR inter-sectoral

**Search #3**

“Health in all policies” OR “healthy public policy” OR “healthy communities” OR “health equity” OR “health inequity” OR “population health” OR “health systems” OR “social determinants of health” OR “health partnership*” OR “urban health” OR “health service*” OR “healthy municipal*” OR “healthy cities” OR “healthy city” OR “intersectoral health” OR “intersectoral model” OR “health authorit*” OR “health sector”

###  Inclusion and Exclusion Criteria 

 Articles were included in the review if they were: (*i*) peer-reviewed, (*ii*) evaluated an intersectoral partnership that occurred between local government and a health partner, (*iii*) reported an outcome (that is, an organisational, social determinant of health or population health outcome) related to change, (*iv*) high-income context,^19^ (*v*) published between (2005-2021), and (*vi*) in English. Articles were excluded if (*i*) they were reviews, study protocols, commentaries, editorials, books, or theses; (*ii*) did not include an evaluative component (that is, the study is reporting on structured interpretation or assessment of the partnership); (*iii*) did not report an outcome related to change; (*iv*) or did not contribute meaningfully to answering the research question, purpose, or objectives. Grey literature was excluded as the review was focused on established, best practice literature on partnerships between local government and health.

###  Study Selection

 Using the inclusion and exclusion criteria, titles and abstracts of all articles retrieved were assessed by an independent reviewer and in line with Cochrane Rapid Review Guidelines^20^ a 20% sample were reviewed by a second reviewer to address risk of selection bias. Where it was unclear whether the selection criteria were met, studies were included for full text review. All full text articles were reviewed by two independent reviewers. Disagreements were resolved by a third reviewer.

###  Data Extraction and Synthesis

 Categorical data from each article (author, year, country, methods, and sample) were extracted. Each paper included in the final synthesis underwent an inductive thematic narrative analysis,^21^ grounded in critical realist methodology.^17^

 Analysis drew on critical realism^17^ identifying factors and conditions that were contingent to the successful facilitation of partnerships between local government and health organisations (eg, contingent factors). Realist analysis searches for necessary and contingent factors. Necessary factors are those that are required to make something happen (oxygen is required when lighting a match, for example). Contingent are those that need to be considered as necessary causal influences but are contingent because they are dependent on being activated (or not) under particular circumstances. A useful analogy for contingent factors is planning to buy a house, where contingent factors need to be identified as ones that might come into play, or may not, as major influences on the outcome.^22^

 Our analysis sought to identify necessary, “contingent” and “contextual” factors that shape partnership outcomes and success. Necessary factors were those identified as essential across contexts for partnerships to work. Contingent factors were characterised as those that influence the likelihood of a successful partnership but may be observable in some but not other contexts; and contextual factors as the context in which events or outcomes related to the partnership occur. This is important to consider when looking at “casual pathways” eg, the sum of factors that create an outcome (in this case a successful or non-successful partnership).

 Data (qualitative or quantitative) related to the facilitation of partnerships between local government and health organisations, and changes or outcomes that resulted from the partnership were coded using NVivo qualitative data analysis software (QSR International Pty Ltd., Version 12, 2018). We defined partnerships as an intersectoral relation, alliance, coalition, informal or formal relationship that includes a local government actor (eg, council, local government or authority) and one or more public health partners (eg, hospital, regional health service), which work to improve health or health services. We defined changes or outcomes as a result of the partnership, to be organisational in nature. Clinical or population health changes/outcomes were analysed as secondary to organisational changes/outcomes.

 The aim was to map these against a “causal pathway” to determine the underlying “mechanisms” that drive partnership outcomes. Where relevant factors were also coded as either a barrier or a facilitator to successful partnerships, enabling us to map factors that contribute to success (or not) across a range of complex and differing contexts. Factors were organised into broader thematic groups and cross-tabulated in NVivo 12 to identify where barriers and facilitators occurred in each thematic group. Thematic groups were discussed among the authors, and further categorized as four mechanisms that shape the success of partnerships between local government and health organisations (Box 2).

**Box 2. Mechanisms Shaping Successful Partnerships Between Local Government and Health Organisations**

**Functional aspects of the partnership:** related to the structure and functioning of the partnership itself.

**Organisational factors impacting the partnership:** related to the structure and culture of the organisations in the partnership.

**Individual factors impacting the partnership:** related to agentic factors surrounding the individuals or actors involved in the partnership eg, personalities, skills.

**External factors impacting the partnership:** related to factors outside of the partnership and organization that have impact on both eg, policy, legislation, local leadership.

Contingent factors in each thematic group were then separated according to whether they were a “barrier” or “facilitator” to successful partnerships, and mapped accordingly across each mechanism. This analysis informed the development of the conceptual framework, which outlines the summation of all factors that lead to successful or un-successful partnerships.

## Results

**Figure 1:**
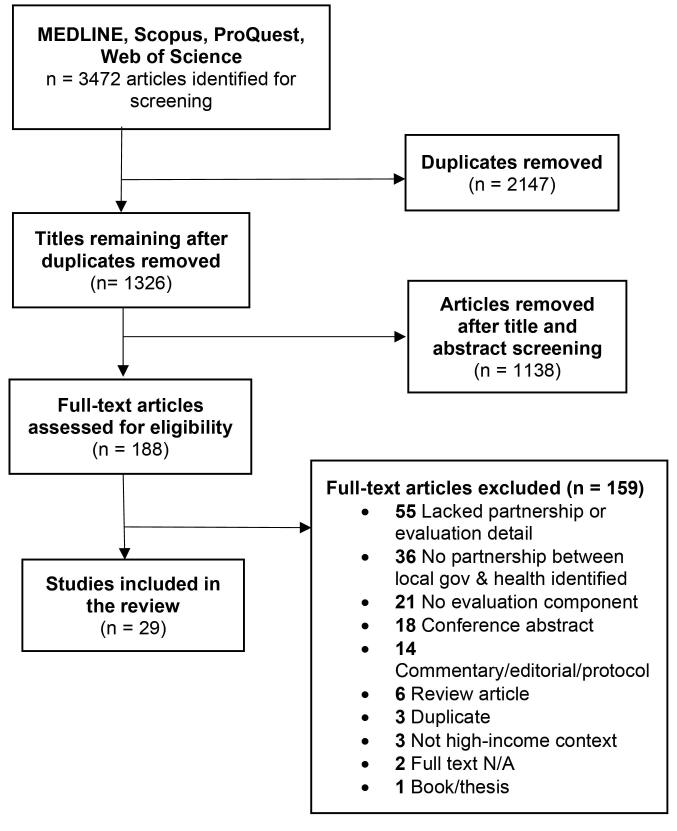
PRISMA Flowchart of Scoping Review Process and Sampling. Abbreviations: PRISMA, Preferred Reporting Items for Systematic Reviews and Meta-Analyses; N/A, not available.

 The database search identified 3472 potential studies. After removal of duplicates 1326 titles and abstracts were screened. Of these, 188 full-text publications were retrieved for consideration. A total of 159 articles were excluded after performing the full text review, leaving 29 articles for inclusion (Figure 1).

 The characteristics of the 29 included articles are outlined in Table 1. The majority of articles reported on partnerships in European countries (n = 8), followed by Canada (n = 7) and the United Kingdom (n = 7), with the remainder situated in Australia (n = 3) and the United States (n = 3). Local government partners broadly included city councils or municipalities, counties (US), local authorities (UK), and local social services or police departments. Health partners included local health departments or districts, hospitals or other health services, public health networks, and First Nations health authorities.

**Table 1:** Characteristics of Included Studies

**First Author (Ref)**	**Year**	**Country**	**Government Actor**	**Health Actor**	**Methodology**	**Participant Type & Sample, N**
Amed^23^	2015	Canada	City council	Hospital	Evaluation framework	2 Communities
Asada^24^	2019	US	County department	Public health institute	Theory of change & longitudinal case study	Key informants (n = 97)
Bachmann^25^	2009	UK	Local authority	NHS; Health authority	Mixed methods: survey & interviews	Key informants, (n = 138)
Chen^26^	2012	US	County department	Local health district	Mixed methods: survey & interviews	Key informants, (n = 148)
Christensen^27^	2019	Denmark	Husum Neighbourhood Renewal & Prevention Centre	Diabetes centre	Qualitative: case study	Health network partners (n = 9); Meeting observations (n = 11)
Dennis^28^	2015	Australia	Exercise & Sports Science Australia	Primary health network	Qualitative: semi-structured interviews	Partnership members (n = 14)
Erens^29^	2019	UK	Local authority	NHS	Quantitative survey	Key informants (n = 3)
Greaux^30^	2020	The Netherlands	Municipality	Ministry of Health	Multi case study	Key informants (n = 153)
Hagen^31^	2015	Norway	Municipality	HiAP (not specified)	Quantitative: cross-sectional	Public health coordinators (n = 332)
Hunter^16^	2012	UK	Local government	NHS (public health)	Qualitative: semi-structured interviews	Key informants (n = 93)
Jabot^32^	2020	Canada	Social services department	Public health organisation(s)	Multi case study	Evaluation of HIA implementation in 2 regions (n = 2)
Jones^33^	2020	UK	Local government	NHS	SROI	Community participants (n = 159)
Kingsnorth^34^	2013	UK	Local government	Public health organisation(s)	PAT	Partnership stakeholders (n = 8)
Kirchhoff^35^	2016	Norway	Local authority	Regional health organisation	Quantitative: survey	Key informants (n = 248)
Kisely^36^	2010	Canada	Local police department	Mental health service; emergency health service	Mixed methods: pre-post, interviews	Patients (n = 295)
Kjelle^37^	2018	Norway	Municipality	Hospital	Qualitative	Key informants (n = 11)
Leurs^38^	2008	The Netherlands	Education department	Regional health organisation	DISC model	Stakeholders (n = 69)
Macleod^39^	2019	Canada	Northern health (5 regional and 1 provincial health authority)	First Nations Health Authority	Qualitative: case study	Stakeholders (n = 122)
Mantoura^40^	2007	Canada	City council	Public health department	Qualitative	Team meetings (n = 12)
Miro^41^	2014	Canada	Ontario Public Health Authority/Regional Health Authorities	Public health network	Quantitative: survey	Stakeholders (n = 12)
Miro^42^	2014	Canada	Municipal & regional planning departments	City health department	Mixed methods: surveys & focus group	Partnership members, (n = 86); Planners and health authority representatives, (n = 8)
Sestoft^43^	2014	Denmark	Local police department	Mental health service	Qualitative: structured interviews & focus groups	Key informants (n = 48); Frontline workers (n = 2)
Storm^44^	2016	The Netherlands	Municipality	Local health districts	Qualitative: documentary analysis, digital questionnaires, semi-structured interviews	Key informants questionnaires (n = 98); interviews (n = 32)
Tooher^45^	2017	Australia	Local government	State health sector	Qualitative: semi-structured interviews	Stakeholders (n = 19)
Tugwell^46^	2011	Australia	City council	Regional health organisation	Evaluation: health impact assessment	Partnership members (n = 17)
Visram^47^	2020	UK	Local authority	Multiple	Comparative case study: mixed methods	Partnership members (n = 79); Stakeholders (n = 23)
Vogel^48^	2007	US	City council	City health department	Retrospective analysis	N/A
Warwick-Giles^49^	2016	UK	Local authority	NHS	Qualitative case studies	Stakeholders (n = 22)
Wistow^50^	2006	UK	Local government	NHS	Case study: mixed methods	Stakeholders questionnaire (n = 18); interviews (n = 16)

Abbreviations: PAT, Partnership Assessment Tool; DISC, Diagnosis of Sustainable Collaboration; SROI, Social return on investment; NHS, National Health Service; N/A, not available; HIA, health impact assessment.

**Figure 2:**
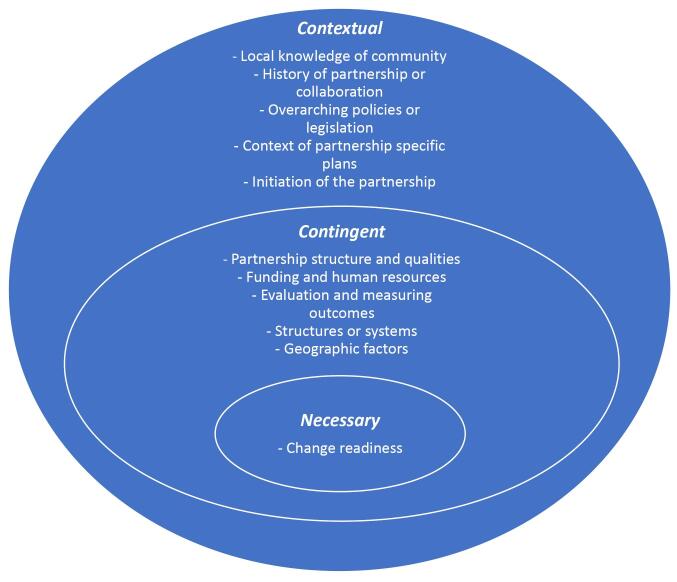
Realist Factors of Partnerships.

 To align with realist approaches, narrative results are presented according to each thematic group of contingent factors identified through the process of inductive analysis. These are: Funding and resources; Partnership qualities; Governance and policy; and Evaluation and measures of success. Contingent factors identified in the included articles were also mapped against identified mechanisms (Box 2), resulting in the framework presented in Table 2. Figure 2 provides an overview of the identified necessary, contingent and contextual factors. There was only one “necessary” factor identified which was “change readiness.” Critical realism defines necessary factors as the conditions that must be in place for a particular outcome to occur. We classified change readiness as necessary, as without it the conditions for a successful partnership cannot occur.

**Table 2:** Factors Contingent to Successful and Unsuccessful Partnerships Between Local Government and Health Organisations in High-Income Countries

**Mechanisms**	**Funding and Resources**	**Partnership Qualities**	**Governance and Policy**	**Examples of Evaluation Measures**	
Functional aspects of the partnership	Funding of joint position^23,24^ or allocated human resources to partnership^25,26,42,48^	Fostering trust, transparency & relationship building between partners (and partner leaders^50^) and their representative actors^16,23,25,27,40,43,45,46,48,49^	Policies to support partnership funding^26^	A shared measurement system with agreement of how success is measured and reported^23,26^	Successful
	Joint commission/pooled budgets^25,37,50^	Clear, open, continuous and equal channels of communication,^23,45,49^ smaller sub-group meetings created safer spaces to talk,^47^ constructive criticism^49^ or debate^50^	Strong formal and informal leadership advocating for the partnership^24,38^	Theory of Change as evaluation tool^24^	
				Identifying action items and plans to follow up at each meeting^23^	
	Willingness to secure external funding to support partnership^37,48^	Collaboratively developed partnership goal,^16,26^ action plan^23,40,50^ or agenda^45,49,50^	Boards representing partnerships are equally representative^25^	Surveying leaders or managers^25^	
	Invest funding in building relationships^16,23,25^	Shared vision, message,^23,49^ enthusiasm,^43^ respect,^47^ and focus on vertical collaboration^16,35^		Service delivery measures (if applicable)^36^	
	Facilitating trust through resource-neutral collaborations^43^	Partnership based on local needs^16,47^ and connections in local communities^23^		PAT (Hardy, 2000)^50^	
		Conflict resolution mechanisms^40,45^		Partnership effectiveness evaluated through networks built & ongoing sustainability^26^	
		Flexible approach^16,40,43,47,48^			
		Interdisplinary^30,40^			
	Lack of sustainable partnership funding^24,26,28,45,48,49^	Unequal power between partner representatives, hierarchical relationships^35,40,47,50^	Strong focus on one aspect or discipline eg, health^27^ or planning^40^ and a segmented approach^32^	Singular focus on improvement in health or social outcomes^25^	Unsuccessful
	Poor management of integrated services^50^	Prioritises structural approaches at the cost of relationship building^16,50^		Long-term goals without achievabla/clear outcomes^27,47^	
	Funding existing initiatives in one partnership sector^23^	Differing expectations of workload^48^		Differing understandings of how to measure effectiveness^26^ or what counts as “evidence”^45^	
	Oversight of partnership costly^48^	Poor management or administration of partnership^26,48^		Resource intensive behaviour-change programs^45^	
Organisational factors impacting partnership	Funding of integrated services between partners^25^	Enabling information creation & sharing between partners^25,40^ eg, knowledge banks stored in shared location^27^	Change readiness and action within organisations to support partnership^24,38,46,48,50^	Policy changes that foster uptake of health and equity^48^	Successful
		Identify areas of overlap between partners/sectors^44^	Organisations with clear systems of management, finance, and information^25^	Health equity as organisational goal eg, Health in All Policies^27^	
		Aligning partnership with organisational core business^45^	Organisational willingness to take risks^50^		
		Shared understanding of the social, organisational and political contexts of the sectors involved^45^	Similarities in organisational culture between partners^48^		
		Inter-organisational capacity building^23-25,46,48^ and mutual learning^46^	Internal communication about policy decisions and directions^45^		
			Power given to joint position^23^		
			Public service rather than profit organisational incentives^25^		
	Lack of common information systems^37^	Historical organisational baggage blocking change^50^	No statutory power within partnership^47^		Unsuccessful
		Lack of understanding and acceptance of interdependence between organisational partners^34^ or “siloed” ways of working^16,29^	Differences in organisational culture,^50^ bureaucratic red tape^16^		
		Self-justification and blame between partners^50^	Lengthy agendas and infrequent meetings with no minutes reported^47^		
		Agenda setting with the goal of conflict avoidance^47^	Unwillingness to share information,^16^ confidentiality concerns^25^		
		Applying internal organisational performance systems to external partners^50^	Current policies and/or unwillingness towards policy change preventing partnership activities^34,48^		
Individual factors impacting partnership		Individual skills aligned to the needs of partnership^25^ eg, leadership,^38^ history, and experience^45^			Successful
		Interpersonal skills – empathy, insight^45^			
		Strong interpersonal communication^45^			
		Relying on individual “champions” or personal relationships to facilitate partnership^45^			Unsuccessful
External factors impacting partnership		Builds on existing partnerships in that social/community context^24^	National or local policy/legislation aligned to or enabled partnership goals^16,25,43,45,47,50^		Successful
			Presence of enthusiastic local leaders^25^		
			Community trust^25^		
			Conflicting sectoral agendas at higher levels of government^45,50^		Unsuccessful
			Party politics or sector reorganisation^16^ preventing partnership engagement^23,25,37,42,49^		
			Poor awareness/prioritisation of health equity among policymakers^44^		

Abbreviation: PAT, Partnership Assessment Tool.

###  Funding and Resourcing of Partnerships

 Funding was identified as a critical factor to partnership functioning and success, including the willingness of partnership actors to secure external funding to sustain their activities.^37,48^ Several studies noted a lack of sustainable funding as a key barrier to the functioning and continuation of the partnership,^24,26,28,45,48,49^ with one study specifying the costly nature of partnership oversight.^48^ Funding existing initiatives in one partnership sector, resulted in less collective action across multiple community sectors, which was exemplified in a Canadian partnership targeting childhood obesity.^23^

 In some studies funding was allocated to: a joint position between the two partners,^23,24^ or pooled budgets were created for partnership activities.^25,37,50^ In other cases existing human resources were allocated to partnership activities.^25,26,42,48^ Overall, sharing funding and resources contributed to positive and functioning partnerships. Investing funding specifically into building relationships was identified as an enabling factor in two studies.^23,25^ Conversely, a partnership in Denmark^43^ was successful in building trust between partners by taking a resource-neutral approach that relied on voluntarism and enthusiasm of partnership actors.

 Organisational factors included the appropriate funding of integrated services that were a part of the partnership. For example, a UK study^25^ sought to integrate local health, education and social services for children (“Children’s Trusts”), whereby adequate funding of integration enabled partnership activities between the National Health Service (NHS) and the local authority.^25^ Another UK study reported how poor management of integrated health and social services, created as part of a partnership between the local government and the NHS created organisational barriers to the partnership.^50^ Similarly a lack of common information systems between a Norwegian municipality and hospital created barriers to implementing mobile radiography services in nursing homes.^37^

###  Qualities of Local Government and Health Partnerships

 The qualities (that is the key factors that define the partnership) within the partnerships reviewed were critical to their functioning and success. Several studies across multiple geographic contexts referred to the qualities of trust and transparency as key to relationship building between partners, including leaders within the partnership^16,50^ or their representative actors.^23,25,27,40,43,45,46,48,49^ Trust building was hampered by unequal power between partner representatives, and hierarchical relationships.^35,40,47,50^ Two UK partnerships between local government and the NHS outlined that prioritising structural approaches (eg, integrating services across sectors) at the cost of “informal” relationship building was detrimental to the partnership.^16,50^

 Clear, open, continuous, and equal channels of communication between partners was also a facilitating factor.^23,45,49^ Specific communication strategies included holding smaller sub-group meetings which created safer spaces to talk,^47^ and encouraging constructive criticism^49^ or open debate on issues.^50^ Other partnership qualities that facilitated successful cooperation included having a shared vision or message,^23,49^ enthusiasm,^43^ respect,^47^ being flexible^16,40,43,47,48^ or interdisciplinary in approach,^30,40^ and focusing on improving vertical collaboration.^16,35^ Such qualities were materialized through collaboratively developed partnership goals,^16,26^ action plans^23,40,50^ or agendas^45,49,50^ which assisted in negotiating, planning and executing ongoing activities and evaluation. Differing expectations of workload between partners, and poor management and administration of the partnership itself, was a barrier to partnership success between counties and local health districts in two US studies.^26,48^ Partnerships in Australia^45^ and Canada^40^ benefited from having conflict resolution mechanisms embedded in their structure. Partnership functions benefited when they were based on local needs^16,47^ and facilitated connections in local communities.^23^

 Enabling qualities related to the broader organisational context included facilitating information creation and sharing between partners.^16,25,40^ For example, a partnership between a neighbourhood renewal initiative and a diabetes centre in Denmark created “knowledge banks” stored in a shared location.^27^ While an Australian study outlined that creating a shared understanding of the social, organisational and political contexts of the sectors involved enabled an “intersectoral point of view” between partners.^45^ A UK study warned against applying internal organisational performance systems to external partners, which inevitably leads to disagreement and confusion.^50^

 Several studies noted the importance of practicing inter-organisational capacity building^23-25,46,48^ and mutual learning between partner actors.^46^ A Dutch^44^ and Australian study^45^ both stated the importance of aligning partnership with the core business of both partner organisations, including identifying areas of overlap to focus on. On the other hand, instances where there was a lack of understanding and acceptance of interdependence between organisational partners^34^ or “siloed” ways of working^29^ created barriers to partnership facilitation. Two UK studies discussed the role of historical organisational baggage, which served to block change being pursued by the partnership.^50^ Actions that reflected self-justification and blame between partners,^50^ or set agendas with the goal of conflict avoidance rather than resolution,^47^ were identified as organisational qualities that did not facilitate successful partnerships.

 The qualities that individual people brought to the partnerships were key to the very functioning of them. Enabling qualities included where individual skills aligned with the needs of the partnership^25^ including strong leadership skills,^38^ a history or experience within the partnership,^45^ strong communication skills, and interpersonal skills such as empathy and insight were considered assets.^45^ Conversely, relying on individuals as the sole drivers of a partnership (eg, champions), or personal relationships as facilitators of the partnership created a barriers to sustainability.^45^

###  Governance and Policy

 National or local policies and legislation were identified as potential facilitating factors for partnerships. In particular, several studies noted the importance of polices which aligned with or enabled partnership goals with partnership success^16,25,43,45,47,50^ as well as policies that supported partnership funding.^26^ However, conflicting sectoral agendas^45,50^ and bureaucratic party politics or sector reorganisation^16,23,25,37,42,49^ were identified as barriers to effective partnerships. One study from the Netherlands noted that poor awareness or a lack of prioritisation of health equity amongst policy makers as detrimental to partnerships.^44^

 Leadership and representation were also facilitating factors for partnerships. Two studies noted strong formal and informal leadership who advocate for the partnership^24,38^ as key to sustaining partnerships. Local context was also noted as a driver of partnerships, in particular the presence of local leaders, establishing community trust and equal representation on the boards of partnerships were beneficial.^25^

 The focus of the partnership can also contribute to its success. When there was a strong focus on one sector or discipline eg, health^27^ or planning^40^ and a segmented approach ^32^, the effectiveness of the partnership was limited. A study from the United Kingdom highlighted that partnerships that were less focused on structures and were informal in nature were more effective.^16^ One study from the United Kingdom found that the strongest motivators for intersectoral action is for public service rather than profit incentives of organisations.^25^

 There were a number of key organisational level factors that were identified as facilitators to effective partnerships. Several studies noted change readiness within organisations to support the partnership as a key driver^24,38,46,48,50^ as was willingness to take risks.^50^ Unwillingness to share information^16,25^ and current policies and/or unwillingness to change policies^34,48^ prevented partnership activities thus limiting the effectiveness of the partnership. Organisations with clear systems of management, finance and information^25^ and effective internal communication about policy decisions and directions^45^ benefited partnerships, one study from the United Kingdom highlighted lengthy agendas and infrequent meeting with no minute recording to hinder the success of a partnership. An American study highlighted the benefits of similarities between organisational culture, specifically the overlap between two government agencies supported the collaboration.^48^ A study from the United Kingdom reiterated this finding, stating that mismatched organisational cultures can lead to major incompatibilities, creating inherent barriers to forming and implementing partnerships.^50^ A Canadian paper stressed the significant amount of power that the “champions” or identified positions can have over the momentum of projects, both in the planning and implementation.^23^

###  Evaluation and Measures of Success in Partnerships

 Evaluation, including accountability and measures of success are important in the functioning and eventual success or failure of a partnership. Several studies noted the need for a shared understanding and agreement of how success should be measured and reported.^23,26^ This included agreeance on what should and should not be considered evidence^45^ whether that be service delivery measures,^36^ network analysis,^26^ integration of health into policies,^48^ surveying leaders of management^25^ or health outcomes.^25,45^ Partnerships where goals had a longer-term focus with no clear or achievable outcomes^27,47^ were not able to demonstrate tangible outcomes. Focusing measures of success on singular health or social outcomes^25^ and the delivery of resource intensive behaviour-change programs^45^ were both identified as barriers to effective partnerships. One UK study highlighted the real challenge in isolating causation of outcomes to the partnerships themselves rather than being an enabler for the delivery of outcomes in a broader context.^16^

 Two papers identified specific tools that could be utilised in measuring the success of partnerships. An American study noted the Theory of Change model to demonstrate structural change,^24^ and a study from the United Kingdom^50^ utilised the Partnership Assessment Tool (PAT) to analyse an integration of health and social care.

 From a project management perspective, one Canadian study suggested identifying action items and plans to follow up at each meeting as an approach to both continue the momentum of the partnership and to ensure it remains accountable.^23^

## Discussion

 Local government is being recognised as increasingly important in its role in progressing population health, well-being and equity.^1,7,51,52^ This scoping review demonstrated the crosscutting factors involved in evaluated partnerships between local government and health sectors, and mapped them in relation to their role in shaping partnership success. The functional, organisational, individual and external mechanisms (eg, Box 2) act as pathways towards partnership outcomes, by identifying which factors shape partnership success (or not).

 Conceptually our realist derived approach^17^ allowed us to tease out a multilayered framework underpinning partnerships (Table 2), providing a roadmap of the various elements that foster or hinder partnerships within and across the organisations involved. Our critical realism based emphasis on necessary, contingent and contextual factors allows a birds eye view of the full range of factors identified in the international (albeit Western) literature that need to be considered when planning successful local government and health partnerships. Contingency planning, is after all, crucial for complex activities that are subject to complicated and often challenging contexts that are outside of the control of those involved. The existing literature on partnerships tends^13-15^ to be focussed on specific contexts, and uninformed by a systematically developed overview of the full range of factors that may be considered when evaluating those partnerships.

 Practically, we found several contingent considerations, as follows, that are likely to be crucial but may be observable in some but not other contexts. These set of factors, here focussed on local government partnerships, are similar to findings from reviews on health in all policies in municipal government and generic partnership functioning.^9,53^

 Functional aspects, related to the structure and functioning of the partnership itself, were the most reported on in the identified studies. Functional aspects reflected the criticality of partnership qualities/components, that spanned the areas of trust, transparency, open and equal communication, collaboration, and a shared vision. In some cases, these qualities were embedded through tangible processes such as shared planning ^23,26,40,50^ or conflict resolution.^40,45^ Clear, open, continuous, and equal channels of communication provided another example, which was achieved through applied processes such as creating appropriately sized groups or spaces for discussion,^47^ and encouraging constructive criticism.^49^

 Trust and equal power sharing were overarching qualities, with their lack being mirrored in examples where partnership challenges occurred, including power imbalances between partners, hierarchical (as opposed to horizontal) relationships,^16,35,40,47,50^ and differing expectations.^48^ These findings point to the importance of defining desired qualities in the establishment of an intersectoral partnership, and ensuring that they are embedded in the plans and processes that structure and action it. This includes funding^23,25^ and supporting qualities such as relationship and rapport building,^16^ that may not align with short term “outcomes” or organisational performance indicators, but are critical to the longer term functioning and success.^39,42^

 Funding structures were also identified as key to partnership functioning, and facilitated trust and relationships were formed between health and local government actors. Partnerships that were not adequately funded lacked sustainability and longevity.^24,26^ Pooled budgets^25,37,50^ funding of joint positions, and shared human resources were key to driving partnership goals and implementation plans forward,^24^ including managerial investments to maintain collaborative work.^30^ Sector funding environments creates challenges for intersectoral partnerships, as associated activities may need to be aligned with existing health or government initiatives, creating restrictions on how the partnership functions.^23^ From an organisational perspective, aligning partnership goals with the core business of each partner, and identifying areas of overlap may serve as a strategic facilitator.^45^ Having similar organisational cultures between partners may also be of benefit.^48^

 The individuals involved in intersectoral partnerships, as actors within organisations, are the gears that drive intersectoral action and implementation.^25,38^ These actor qualities have been described as leadership characteristics in intersectoral partnerships, where leaders galvanise creative resources. The personalities and skills of organisational actors are equally as important as the roles carved out for them. For example, certain individuals who may champion partnership activities, or rely on personal relationships to facilitate progress may achieve short term success, but challenge longer-term sustainability (eg, beyond their involvement).^45^ Ensuring that individuals involved have the skills needed to achieve the goals of the partnership is key, in addition to strong interpersonal communication skills given the collaborative nature of such work.^45^

 Political and legislative environments are also integral, yet reach beyond the control of people and organisations. Our review identified that aligning partnership goals with existing policies and legislation is key to success.^16,25,43,45,47,50^ The literature on health in all policies similarly tends to exhort the importance of centralised mandates for local action. Political will^32^ or buy in^23^ were in some contexts vital to facilitating intersectoral relationships and securing funding. Conversely, bipartisan politics and sector reorganisation has served as a consistent challenge to intersectoral partnerships, creating barriers to achieve partnership alignment with sectoral agendas.^16,50^

 This paper adds a specific local government focussed lens to the burgeoning literature on intersectoral partnerships^53-57^ with a health focus. Our review found that intersectoral partnerships between local government and health sectors are complex, multifaceted, and require the alignment of certain qualities, contextual, and environmental factors to be successful. Some factors fall within the control of the actors and organisations involved in intersectoral partnerships, while other factors such as that related to politics and legislation may not. For this reason, ensuring a strong understanding of the social, organisational, and political contexts of the sectors involved, and aligning partnerships with these contexts is crucial.^45^ Our framework (Table 2) provides a breadth of factors at multiple layers to consider, that will enable partnership designers to contemplate the elements needed to achieve successful partnerships, and evaluators to develop and implement detailed theories of change in their applied contexts.

## Limitations

 This scoping review was limited to peer-review articles, and therefore may have missed potentially relevant information in grey literature articles, books and theses. There were limitations in capturing and presenting contextual variation due to the geographic breadth, and contextual depth of the identified studies. However, our focus on evaluations helped navigate the breadth of literature. The results are presented as an overview and therefore some (contingent, as per the definition) factors may be more or less activated in different contexts. As per scoping review methods,^58^ presenting an overview of the breadth of studies meant that the included literature was not assessed on quality in terms of bias, validity or generalisability further, a protocol for this review was not registered.

## Conclusion

 Local government is the layer of government closest to community health. Local government agencies develop and implement policies, plans and services that have direct impact on the health of local people. This paper has outlined the core factors in the international literature that inform successful partnerships between health and local government agencies. By drawing out factors that have been identified as influential across contexts, we have provided a sophisticated framework to consider when developing and evaluating health and local government partnerships.

## Ethical issues

 Not applicable.

## Competing interests

 Authors declare that they have no competing interests.

## Funding

 This work was supported by South Western Sydney Local Health District Population Health as part a broader research project, developing a theory of change to underpin an evaluation of partnerships (case studies) between health and local government in Sydney, Australia.

## References

[1] Lawless A, Lane A, Lewis FA, Baum F, Harris P (2017). Social determinants of health and local government: understanding and uptake of ideas in two Australian states. Aust N Z J Public Health.

[2] Harris E, Wills J (1997). Developing healthy local communities at local government level: lessons from the past decade. Aust N Z J Public Health.

[3] Pierre J (1999). Models of urban governance: the institutional dimension of urban politics. Urban Aff Rev Thousand Oaks Calif.

[4] Peters BG, Pierre J. Urban governance. In: John P, Mossberger K, Clarke SE. The Oxford Handbook of Urban Politics. Oxford University Press; 2012.

[5] Healey P, Cars G, Madanipour A, De Magalhaes C. Transforming governance, institutionalist analysis and institutional capacity. In: Urban Governance, Institutional Capacity and Social Milieux. Routledge. 2017:6-28.

[6] Lowndes V, Leach S (2004). Understanding local political leadership: constitutions, contexts and capabilities. Local Gov Stud.

[7] de Leeuw E, Polman L (1995). Health policy making: the Dutch experience. Soc Sci Med.

[8] Guglielmin M, Muntaner C, O’Campo P, Shankardass K (2018). A scoping review of the implementation of health in all policies at the local level. Health Policy.

[9] Van Vliet-Brown CE, Shahram S, Oelke ND (2018). Health in all policies utilization by municipal governments: scoping review. Health Promot Int.

[10] Schultz S, Zorbas C, Peeters A, Yoong S, Backholer K (2023). Strengthening local government policies to address health inequities: perspectives from Australian local government stakeholders. Int J Equity Health.

[11] Browne GR, Davern M, Giles-Corti B (2019). ‘Punching above their weight’: a qualitative examination of local governments’ organisational efficacy to improve the social determinants of health. Aust N Z J Public Health.

[12] Browne GR, Davern MT, Giles-Corti B (2016). An analysis of local government health policy against state priorities and a social determinants framework. Aust N Z J Public Health.

[13] Holt DH, Frohlich KL, Tjørnhøj-Thomsen T, Clavier C (2017). Intersectoriality in Danish municipalities: corrupting the social determinants of health?. Health Promot Int.

[14] Hagen S, Torp S, Helgesen M, Fosse E (2017). Promoting health by addressing living conditions in Norwegian municipalities. Health Promot Int.

[15] Fosse E, Helgesen MK, Hagen S, Torp S (2018). Addressing the social determinants of health at the local level: opportunities and challenges. Scand J Public Health.

[16] Hunter D, Perkins N (2012). Partnership working in public health: the implications for governance of a systems approach. J Health Serv Res Policy.

[17] Bhaskar R. A Realist Theory of Science. Routledge; 2013.

[18] Tricco AC, Lillie E, Zarin W (2018). PRISMA extension for scoping reviews (PRISMA-ScR): checklist and explanation. Ann Intern Med.

[19] United Nations. World Economic Situation and Prospects 2014. United Nations Publications; 2014.

[20] Garritty C, Gartlehner G, Nussbaumer-Streit B (2021). Cochrane Rapid Reviews Methods Group offers evidence-informed guidance to conduct rapid reviews. J Clin Epidemiol.

[21] Glaser BG, Strauss AL. The Discovery of Grounded Theory: Strategies for Qualitative Research. Routledge; 2009.

[22] Harris P. Illuminating Policy for Health: Insights from a Decade of Researching Urban and Regional Planning. Springer; 2022.

[23] Amed S, Naylor PJ, Pinkney S (2015). Creating a collective impact on childhood obesity: lessons from the SCOPE initiative. Can J Public Health.

[24] Asada Y, Gilmet K, Welter C, Massuda-Barnett G, Kapadia DA, Fagen M (2019). Applying theory of change to a structural change initiative: evaluation of model communities in a diverse county. Health Educ Behav.

[25] Bachmann MO, O’Brien M, Husbands C (2009). Integrating children’s services in England: national evaluation of children’s trusts. Child Care Health Dev.

[26] Chen LW, Roberts S, Xu L, Jacobson J, Palm D (2012). Effectiveness and challenges of regional public health partnerships in Nebraska. J Public Health Manag Pract.

[27] Christensen JH, Bloch P, Møller SR (2019). Health in all local policies: lessons learned on intersectoral collaboration in a community-based health promotion network in Denmark. Int J Health Plann Manage.

[28] Dennis S, Hetherington SA, Borodzicz JA, Hermiz O, Zwar NA (2015). Challenges to establishing successful partnerships in community health promotion programs: local experiences from the national implementation of healthy eating activity and lifestyle (HEAL^TM^) program. Health Promot J Austr.

[29] Erens B, Wistow G, Mays N (2020). Can health and social care integration make long-term progress? Findings from key informant surveys of the integration Pioneers in England. J Integr Care.

[30] Grêaux KM, de Vries NK, Bessems K, Harting J, van Assema P (2021). Does partnership diversity in intersectoral policymaking matter for health promoting intervention packages’ composition? A multiple-case study in the Netherlands. Health Promot Int.

[31] Hagen S, Helgesen M, Torp S, Fosse E (2015). Health in all policies: a cross-sectional study of the public health coordinators’ role in Norwegian municipalities. Scand J Public Health.

[32] Jabot F, Tremblay E, Rivadeneyra A, Diallo TA, Lapointe G (2020). A comparative analysis of health impact assessment implementation models in the regions of Montérégie (Québec, Canada) and Nouvelle-Aquitaine (France). Int J Environ Res Public Health.

[33] Jones C, Hartfiel N, Brocklehurst P, Lynch M, Edwards RT (2020). Social return on investment analysis of the health precinct community hub for chronic conditions. Int J Environ Res Public Health.

[34] Kingsnorth R (2013). Partnerships for health and wellbeing. J Integr Care.

[35] Kirchhoff R, Ljunggren B (2016). Aspects of equality in mandatory partnerships - from the perspective of municipal care in Norway. Int J Integr Care.

[36] Kisely S, Campbell LA, Peddle S (2010). A controlled before-and-after evaluation of a mobile crisis partnership between mental health and police services in Nova Scotia. Can J Psychiatry.

[37] Kjelle E, Lysdahl KB, Olerud HM, Myklebust AM (2018). Managers’ experience of success criteria and barriers to implementing mobile radiography services in nursing homes in Norway: a qualitative study. BMC Health Serv Res.

[38] Leurs MT, Mur-Veeman IM, van der Sar R, Schaalma HP, de Vries NK (2008). Diagnosis of sustainable collaboration in health promotion - a case study. BMC Public Health.

[39] MacLeod MLP, Hanlon N, Reay T, Snadden D, Ulrich C (2019). Partnering for change. J Health Organ Manag.

[40] Mantoura P, Gendron S, Potvin L (2007). Participatory research in public health: creating innovative alliances for health. Health Place.

[41] Miro A, Perrotta K, Evans H (2014). Building the capacity of health authorities to influence land use and transportation planning: lessons learned from the Healthy Canada by Design CLASP Project in British Columbia. Can J Public Health.

[42] Miro A, Kishchuk NA, Perrotta K, Swinkels HM (2014). Healthy Canada by Design CLASP: lessons learned from the first phase of an intersectoral, cross-provincial, built environment initiative. Can J Public Health.

[43] Sestoft D, Rasmussen MF, Vitus K, Kongsrud L (2014). The police, social services and psychiatry cooperation in Denmark--a new model of working practice between governmental sectors A description of the concept, process, practice and experience. Int J Law Psychiatry.

[44] Storm I, den Hertog F, van Oers H, Schuit AJ (2016). How to improve collaboration between the public health sector and other policy sectors to reduce health inequalities? - A study in sixteen municipalities in the Netherlands. Int J Equity Health.

[45] Tooher R, Collins J, Braunack-Mayer A (2017). Intersectoral collaboration to implement school-based health programmes: Australian perspectives. Health Promot Int.

[46] Tugwell A, Johnson P (2011). The Coffs Harbour ‘our living city settlement strategy’ health impact assessment. Environ Impact Assess Rev.

[47] Visram S, Hunter DJ, Perkins N (2021). Health and wellbeing boards as theatres of accountability: a dramaturgical analysis. Local Gov Stud.

[48] Vogel A, Ransom P, Wai S, Luisi D (2007). Integrating health and social services for older adults: a case study of interagency collaboration. J Health Hum Serv Adm.

[49] Warwick-Giles L, Coleman A, Checkland K (2016). Co-owner, service provider, critical friend? The role of public health in clinical commissioning groups. J Public Health (Oxf).

[50] Wistow G, Waddington E (2006). Learning from doing: implications of the Barking and Dagenham experience for integrating health and social care. J Integr Care.

[51] Freitas Â, Rodrigues TC, Santana P (2020). Assessing urban health inequities through a multidimensional and participatory framework: evidence from the EURO-HEALTHY project. J Urban Health.

[52] Lee CB, Huang NC, Kung SF, Hu SC (2021). Opportunity for HiAP through a Healthy Cities initiative in Taiwan: a multiple streams analysis. Health Promot Int.

[53] Corbin JH, Jones J, Barry MM (2018). What makes intersectoral partnerships for health promotion work? A review of the international literature. Health Promot Int.

[54] de Leeuw E (2022). Intersectorality and health: a glossary. J Epidemiol Community Health.

[55] Dowling B, Powell M, Glendinning C (2004). Conceptualising successful partnerships. Health Soc Care Community.

[56] Mitchell SM, Shortell SM (2000). The governance and management of effective community health partnerships: a typology for research, policy, and practice. Milbank Q.

[57] Jaques K, Haigh F, Zapart S (2023). Inter-sectoral policy partnerships: a case study of South Western Sydney’s Health and Housing Partnership. Int J Hous Policy.

[58] Munn Z, Peters MDJ, Stern C, Tufanaru C, McArthur A, Aromataris E (2018). Systematic review or scoping review? Guidance for authors when choosing between a systematic or scoping review approach. BMC Med Res Methodol.

